# Association, Haplotype, and Gene-Gene Interactions of the HPA Axis Genes with Suicidal Behaviour in Affective Disorders

**DOI:** 10.1155/2013/207361

**Published:** 2013-11-27

**Authors:** Anna Leszczyńska-Rodziewicz, Aleksandra Szczepankiewicz, Joanna Pawlak, Monika Dmitrzak-Weglarz, Joanna Hauser

**Affiliations:** ^1^Department of Psychiatric Genetics, Poznan University of Medical Sciences, Ulica Szpitalna 27/33, 60-572 Poznań, Poland; ^2^Laboratory of Molecular and Cell Biology, Department of Pulmonology, Paediatric Allergy and Clinical Immunology, Poznan University of Medical Sciences, Ulica Szpitalna 27/33, 60-687 Poznań, Poland

## Abstract

Family twin and adoption studies have noted the heritability of specific biological factors that influence suicidal behaviour. Exposure to stress is one of the factors that strongly contribute to suicide attempts. The biological response to stress involves the hypothalamic-pituitary-adrenal axis (HPA). Therefore, we found it interesting to study polymorphisms of genes involved in the HPA axis (*CRHR1, NR3C1,* and *AVPBR1*). The study was performed on 597 patients, 225 of whom had a history of suicide attempts. We did not observe any significant differences in the studied polymorphisms between the group of patients with a history of suicide attempts and the control subjects. Our haplotype analysis of the *AVPR1b* gene revealed an association between the GCA haplotype and suicide attempts; however, this association was not significant after correcting for multiple testing. We did not observe any other association in haplotype and MDR analysis. We report here a comprehensive analysis of the HPA axis genes and a lack of association for genetic variations regarding the risk of suicide attempts in affective disorder patients. Nonetheless, the inconsistencies with the previously published results indicate the importance of the further investigation of these polymorphisms with respect to the risk of suicide attempts.

## 1. Introduction

According to the WHO, approximately 1 million people commit suicide each year. Therefore, suicide is considered a major public health problem. The causes of suicidal behaviour and suicide attempts are complex in that both genetic and environmental factors can play a role. Exposure to an acute or chronic stress accompanied by psychiatric conditions, including substance abuse, depressed mood, anxiety, and also psychotic features, can lead to suicide attempts.

Family twin and adoption studies have noted the heritability of these features, suggesting that there is a specific biological influence on suicidal behaviour. Genes containing different variants, interindividual differences in psychology, and related stress resilience may cause a vulnerability to suicidal behaviour [1], as well as age, sex and the history of the trauma. A majority of the genetic studies have involved serotonin-related genes because levels of serotonin and its metabolites are thought to play a role in suicidal behaviour. Meta-analyses have confirmed an association between suicidal behaviour and 5HTT, (serotonin transporter), tryptophan hydroxylase (TPH1), and brain-derived neurotropic factor (BDNF) expression, as well as the expression of tyrosine kinase receptor type 2 (NTRK2) [2, 3].

The biological response to stress involves the hypothalamic-pituitary-adrenal axis (HPA), and prolonged stress may change its function. These changes can be marked by altered HPA activity (high or low cortisol levels) or reactivity (reduced or increased feedback regulation). These HPA disturbances are also thought to be partially predictive of suicidal behaviour [4–6] and are regarded as an endophenotype of suicidal behaviour [5]. In particular, AVP receptor upregulation may be critical for sustaining corticotropic responsiveness during chronic stress or depression [7]. Jokinen et al. suggested that dexamethasone suppression test (DST) nonsuppression may be a biological suicide predictor in depressed male suicide attempters [8, 9]. Those patients may be genetically predisposed to react with high levels of HPA activity in response to a low stress and may experience a chronic state of HPA activation [10, 11]. This reaction occurs most likely via altered *CRHR1* (the gene controlling activation of HPA axis) expression and functionality. Recently, Merali and colleagues found that *CRHR1* mRNA expression was changed in the frontal cortices of suicide victims [12]. Hyperactivity of the HPA axis predicts a worse treatment outcome [13], and Binder et al. suggested that dysregulation of the HPA axis may even predict a nonresponse to antidepressant treatment particularly among males [14]. Both low CSF5-HIAA and DST nonsuppression contribute to a fourfold increased suicide risk in depressed subjects in a meta-analysis performed by Mann and Currier [5]. Some studies have reported no association between the DST test and individual susceptibility to suicide [15]. McGowan et al. observed epigenetic modification of the glucocorticoid receptor gene as a function of childhood abuse in the brains of suicide completers [16]. Thus, it is possible that HPA axis dysfunction is not linearly related to suicide risk [17]. Therefore, it would be interesting to study the polymorphisms of genes encoding receptors and transporters involved in the regulation of HPA axis function. Taking into account the limited number of genetic studies that have been published previously on the association between HPA axis genes and the history of suicide attempts, we aimed in the present study to investigate the possible association between *NR3C1*, *CRHR1*, and *AVPR1b* gene polymorphisms and suicidal behaviour in unipolar (UP) and bipolar (BP) patients.

## 2. Materials and Methods

### 2.1. Patients

We included 597 patients (367 female, 230 male), aged 18–84 (mean = 47, SD = 14) who met DSM-IV criteria for bipolar disorder (391 BPI, 104 BPII) or recurrent depression (*n* = 102) and were living in the Wielkopolska region of Poland. The diagnosis was established using the Structured Clinical Interview for DSM-IV Axis I Disorders (SCID-I) [18]. Among patients with BP disorder, 197 people had a history of suicide attempt(s); among the UP patients, 28 people had a history of suicide attempt(s). The range of duration for disease in our group was 1–54 years (mean 15 years, SD = 11). Informed consent was obtained from each patient after the nature of the procedure had been fully explained to them.

### 2.2. Control Group

The control group consisted of 712 subjects. Control subjects were recruited from a group of healthy volunteers, blood donors and hospital staff, and students of the University of Medical Sciences in Poznan and the Clinical Neuropsychology Unit, Collegium Medicum Bydgoszcz. Only 40% of the control group were psychiatrically screened using the Polish version of the M.I.N.I. Plus scale to exclude individuals with any serious mental health problems. The mean age was 37.7, SD = 12.5. The control group was matched with age and sex. The study was approved by our local ethics committee.

### 2.3. Genotyping

DNA was extracted from using the salting out method [19]. SNP selection was carried out under the following criteria: functionality (in experimental functional studies), high frequency (MAF > 0.05), indication as a tag SNP in HapMap, or previously reported associations with psychiatric disorders (both positive and negative findings). The SNPs chosen included both coding regions of known functionality and noncoding regions (introns, UTRs) that could affect gene regulation. The* NR3C1*, *CRHR1*, and *AVPR1b* polymorphisms were genotyped using TaqMan SNP Genotyping assays (Applied Biosystems) and TaqMan Genotyping Master Mix. The list of SNPs analysed and the ID numbers of the TaqMan assays were used according to previously described findings [20]. For each reaction plate, nontemplate controls (water) and genomic control DNA samples were included. To check for genotyping accuracy control TaqMan SNP genotyping assay was performed, 15% of randomly chosen samples from both groups, and identical genotypes were identified in all repeated samples. The clinical status of the subjects was not known during genotyping. The genotyping success rates were between 96.03% and 98.97%. The total number of genotyped patients differs on the different SNPs due to genotyping errors and therefore exclusions from further studies.

### 2.4. Statistical Analysis

Two-tailed Pearson's chi-squared (*χ* ^2^) test and Fisher's exact test were, respectively, used to test differences in the genotypic and allelic distribution between the groups of patients and the control subjects. Two-tailed power analysis was also performed. Calculations were performed using Statistica version 9.0. Odds ratios were calculated using 2 × 2 contingency tables by using Fisher's exact test in a demo version of GraphPad InStat 3 software. Linkage disequilibrium (LD) between the *CRHR1*, *AVPR1b*, and *NR3C1* polymorphisms was examined by pairwise comparisons of *r* ^2^ and *D*′ using Haploview version 4.1 [21]. Corrections for multiple testing were performed for multiple comparisons in the haplotype analysis and were completed for 10,000 permutations.

Higher-order gene-gene interactions among the tested SNPs were analysed using the nonparametric and genetic model-free multifactor dimensionality reduction (MDR) approach (v.2.0 beta 8.3). All interactions were tested using 10-fold cross-validation in an exhaustive search considering all possible SNP combinations. The model with the highest testing balance accuracy and cross-validation consistency of >5 out of 10 was selected as the “best model.” Statistical significance was determined using a 1000-fold permutation test (MDR permutation testing module, v.1.0 beta 2). The software is available online (http://www.epistasis.org/).

The power to detect an association for an odds ratio of 1.5 for our sample was about 80% for each SNP.

## 3. Results

### 3.1. HWE Analysis

The genotype distributions were in Hardy-Weinberg equilibrium, except the following polymorphisms: rs16940655 of *CRHR1*, rs10052957, and rs258813 of *NR3C1*, and rs28632197 of *AVPBR1* in the group of the patients and the control group. We did not include those polymorphisms into the further analysis.

### 3.2. Association Analysis

We did not observe any significant differences in the studied polymorphisms between the group of patients with a history of suicide attempts and the control subjects or in genotype distribution (results shown in Tables 1, 2, and 3, 0 = controls, 1 = suicidal patients) or allele frequencies (data not shown). Analysis stratified by gender also did not reveal any statistically significant differences between patients with suicide attempts and the control group (data not shown). We also compared the bipolar patients with the history of suicide attempts versus bipolar patients without suicide attempts in the past, but no significance was found between the two groups (data not shown).

### 3.3. Haplotype Analysis

**Figure 1:**
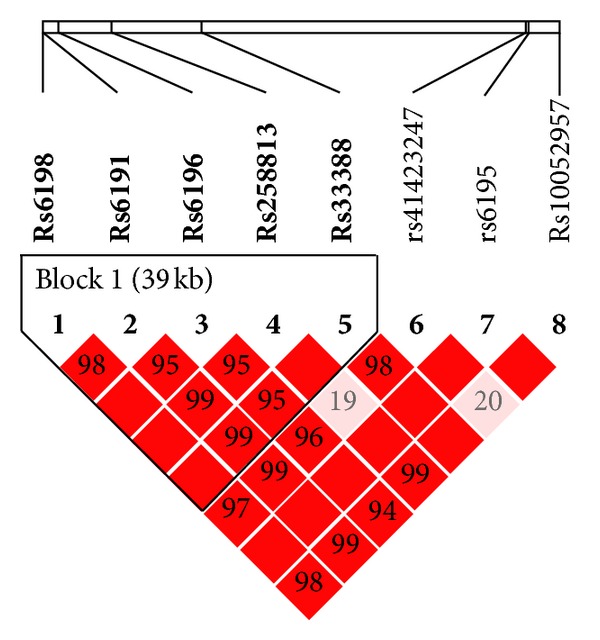
Relative positions and LD estimates between *NR3C1* polymorphisms in the analyzed population. Coloured squares correspond to *D*′ values with numerical estimates given within the squares.

Linkage disequilibrium analysis of the three analysed genes revealed strong linkage between the polymorphisms. For the *NR3C1* gene, we observed linkage for 5 out of 8 studied polymorphisms grouped in one haplotype block (*D*′ = 1.0, LOD = 39.11, *r* ^2^ = 0.162) (Figure 1).

However, none of the haplotypes were significantly more frequent in the group of patients with suicide attempts compared to the control group (Table 4).

**Figure 2:**
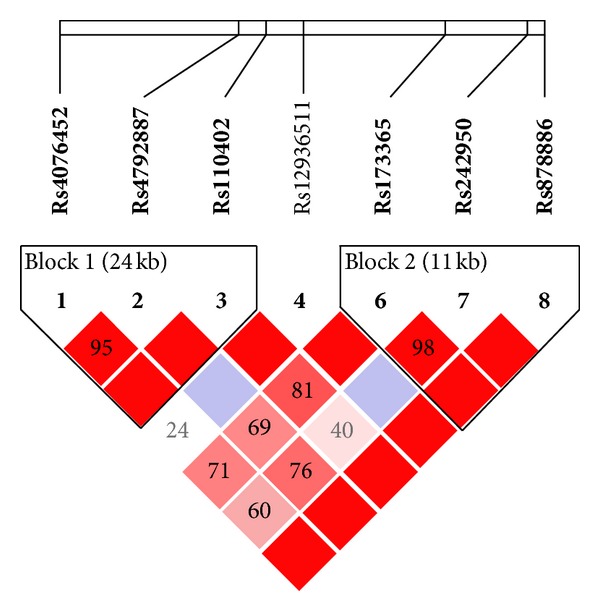
Relative positions and LD estimates between *AVPR1b* polymorphisms in the analyzed population. Coloured squares correspond to *D*′ values with numerical estimates given within the squares.

For the *CRHR1* gene, we observed linkage in 6 out of 7 polymorphisms grouped in two haplotype blocks: rs4076452, rs4792887, are rs110402 in one block (*D*′ = 1.0, LOD = 37.66, *r* ^2^ = 0.216) and rs173365, rs242950, and rs878886 in the other block (*D*′ = 1.0, LOD = 54.9, *r* ^2^ = 0.285) (Figure 2).

Our haplotype analysis did not reveal any significant differences in the haplotype frequencies of the two blocks between patients with suicide attempts and control subjects (Table 5).

**Figure 3:**
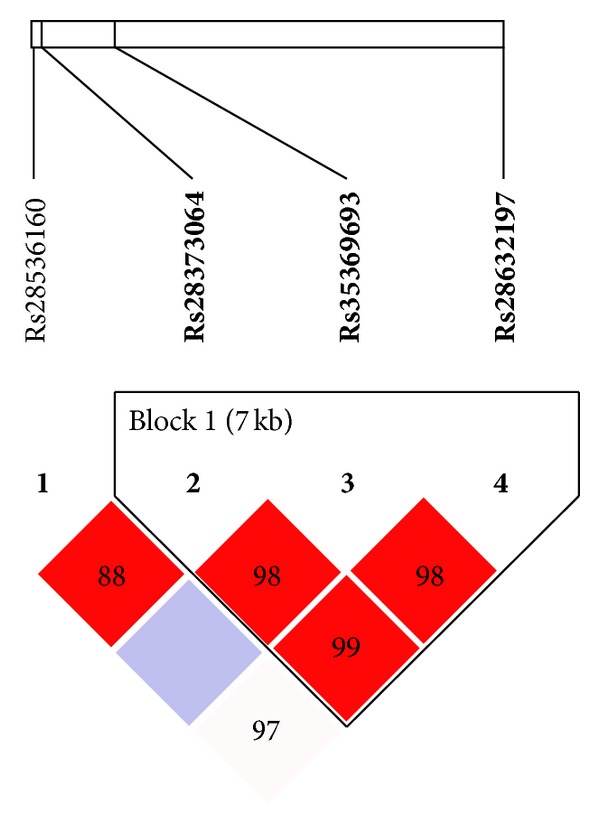
Relative positions and LD estimates between *CRHR1* polymorphisms in the analyzed population. Coloured squares correspond to *D*′ values with numerical estimates given within the squares.

For the *AVPR1b *gene, we observed linkage disequilibrium for 3 out of 4 analysed polymorphisms grouped in one haplotype block (*D*′ = 0.98, LOD = 45.56, *r* ^2^ = 0.302) (Figure 3).

In our haplotype analysis, we observed an association between the GCA haplotype and suicide attempts; however, this association was not significant after correction for multiple testing (*P* value of 0.130 after 10,000 permutations) (Table 6).

### 3.4. Gene × Gene Interaction Analysis

The results of our exhaustive MDR analysis evaluating combinations of all tested SNPs are summarised in Table 7.

The best combination of possibly interactive polymorphisms in predicting suicidal attempts was observed in the 4-locus model. However, no significance was observed for this combination (the testing balanced accuracy for this 4-locus model was 90%, cross-validation consistency was 5/10 (50%), and an empiric *P* value of 0.589 is based on 1000-fold permutations). Similarly, other combinations of the analysed polymorphisms also did not reach statistical significance in predicting susceptibility to an increased risk of suicide attempts.

## 4. Discussion

The results of our study suggested a possible association between the haplotype of the *AVPR1b *gene and suicide attempts, which did not reach significance after multiple testing correction. We did not find any interactions for genotypes or for the alleles of the studied polymorphisms in the group of suicidal patients. Also Dempster et al. suggested the involvement of the *AVPR1b* gene polymorphisms in the etiology of childhood onset mood disorders particularly in females [22]. Ben-Efraim et al. found association between the high Beck scale results in patients with suicide attempts and rs33990840 and a major 6-SNP haplotype of *AVPR1b* gene [23]. Previously, we reported the association between the rs28536160 polymorphism of the *AVPR1b* gene and rs1293651 of the *CRHR1 *gene and bipolar patients with psychotic features [20]. In this study, we did not find association between polymorphisms of *CRHR1 *gene and suicidal patients although Wasserman et al. identified a *CRHR1 *SNP that showed an association in the suicide attempters exposed to low-medium stress and a relationship between neurotic personality traits and suicidality. The genetic variation in the *TBX19* gene (a regulator of the HPA axis) was observed by Wasserman et al. [24, 25]. In suicidal males, they found an association between the T and A alleles of SNPs rs4792287 and rs110402 and the T allele of rs12936511 of the *CRHR1* gene. Polymorphisms in the *CRHR1* gene have also been associated with depression and the treatment efficiency of depression [26–29]. Papiol et al. showed associations of the A allele of the *CRHR1* rs110402 SNP with hte age of onset and with seasonal pattern of major depression [29]. Bradley et al. reported that the T and G alleles of the rs4792887 and rs110402 *CRHR1* SNPs were linked with depression [26]. In the study performed by De Luca et al. an association between haplotype variation at the CRHR2 locus and suicidal behaviour was observed [30]. Interestingly, they also found interactions between polymorphisms of *CRHR1 *gene in suicidal schizophrenic patients [31]. We did not find an association between the polymorphisms of the *NR3C1*, *CRHR1,* and *AVPBR1* genes and the whole group of affective disorder patients (data not published). However, in the study performed by Szczepankiewicz et al. [32], an association between *NR3C1* polymorphisms and depression was found.

The main limitations of our study that might have yielded false-positive or- negative results include the following: the limited sample size of the patient group (*n* = 225), the fact that only 40% of the control group had been screened for psychiatric disorders, and the lack of Hardy-Weinberg equilibrium for the four analysed SNPs. However, taking into consideration the lifetime prevalence of psychiatric disorders and the power of the studied polymorphisms (>80%), it seems unlikely that these factors affected our results.

## 5. Conclusions

We report here a comprehensive analysis of HPA axis genes and a lack of association of genetic variation with the risk of suicide attempts in affective disorder patients. Nonetheless, the inconsistencies with the previously published associations indicate the importance of further investigation of those polymorphisms with respect to the risk of suicide attempts.

## Figures and Tables

**Table 1:** Polymorphisms in *GR* (*NR3C1*) gene (genotypes).

rs41423247	CC		CG		GG		*P*
	*n*	%	*n*	%	*n*	%	
0	65	13.03	239	47.9	195	39.8	0.55
1	27	13.5	87	43.5	86	43	

rs6195	CC		CT		TT		*P*
	*n*	%	*n*	%	*n*	%	

0	450	88.93	56	11.07	0	0	0.28
1	177	88.5	22	11.0	1	0.5	

rs6198	CC		CT		TT		*P*
	*n*	%	*n*	%	*n*	%	

0	14	2.8	116	23.2	370	74	0.33
1	3	1.53	54	27.55	139	70.92	

rs6191	AA		AC		CC		*P*
	*n*	%	*n*	%	*n*	%	

0	136	27.15	244	48.7	121	24.15	0.72
1	57	29.38	88	45.36	49	25.26	

rs6196	AA		AG		GG		*P*
	*n*	%	*n*	%	*n*	%	

0	366	72.05	129	25.39	13	2.56	0.2
1	146	73	53	26.5	1	0.5	

rs33388	AA		AT		TT		*P*
	*n*	%	*n*	%	*n*	%	

0	138	27.27	245	48.42	123	24.31	0.84
1	58	29.15	92	46.23	49	24.62	

0: control. 1: suicidal patients.

**Table 2:** Polymorphisms in *AVPR1b* gene (genotypes).

rs28536160	CC		CT		TT		*P*
	*n*	%	*n*	%	*n*	%	
0	2	0.4	73	14.48	429	85.12	
1	0	0	22	11	178	89	0.31

rs28373064	AA		AG		GG		*P*
	*n*	%	*n*	%	*n*	%	

0	349	68.84	139	27.42	19	3.75	
1	133	66.5	60	30	7	3.5	0.78

rs35369693	CC		CG		GG		*P*
	*n*	%	*n*	%	*n*	%	

0	1	0.2	51	10.39	439	89.41	0.09
1	0	0	31	16.15	161	83.85	

0: control. 1: suicidal patients.

**Table 3:** Polymorphisms of *CRHR1* gene (genotypes).

rs4076452	CC		CG		GG		*P*
	*n*	%	*n*	%	*n*	%	
0	11	2.54	142	32.79	280	64.67	0.48
1	5	3.09	45	27.78	112	69.14	

rs12936511	CC		CT		TT		*P*
	*n*	%	*n*	%	*n*	%	

0	394	91.8	34	7.93	1	0.23	
1	149	91.98	12	7.41	1	0.62	0.75

rs4792887	CC		CT		TT		*P*
	*n*	%	*n*	%	*n*	%	

0	332	77.2	94	21.86	4	0.93	
1	130	79.75	32	19.63	1	0.61	0.77

rs242950	CC		CT		TT		*P*
	*n*	%	*n*	%	*n*	%	

0	325	75.4	99	22.97	7	1.62	0.81
1	126	77.78	34	20.99	2	1.23	

rs878886	CC		CG		GG		*P*
	*n*	%	*n*	%	*n*	%	

0	304	70.2	118	27.25	11	2.5	0.5
1	106	65.4	51	31.48	5	3.09	

rs173365	AA		AG		GG		*P*
	*n*	%	*n*	%	*n*	%	

0	78	18.01	206	47.58	149	34.41	0.9
1	28	17.18	77	47.24	58	35.58	

rs110402	AA		AG		GG		*P*
	*n*	%	*n*	%	*n*	%	

0	98	22.9	216	50.47	114	26.64	
1	39	24.07	87	53.7	36	22.22	0.5

0: control. 1: suicidal patients.

**Table 4:** Comparison of haplotype frequencies for the analysed *NR3C1* polymorphisms between patients with suicide attempts and the control group.

Haplotype	Haplotype frequency	Case : control ratio	*χ* ^2^	*P*
Block 1				
TCAGT	0.478	0.475 : 0.479	0.015	0.901
TAAGA	0.222	0.218 : 0.224	0.072	0.788
CAAAA	0.150	0.170 : 0.142	1.711	0.190
TAGAA	0.144	0.135 : 0.147	0.332	0.564

**Table 5:** Comparison of haplotype frequencies for the analysed *CRHR1 *polymorphisms between patients with suicide attempts and the control group.

Haplotype	Haplotype frequency	Case : control ratio	*χ* ^2^	*P*
Block 1				
GCA	0.489	0.510 : 0.481	0.755	0.385
GCG	0.323	0.321 : 0.323	0.0050	0.943
CTG	0.110	0.104 : 0.112	0.166	0.683
CCG	0.074	0.065 : 0.077	0.487	0.485

Block 2				
GCC	0.584	0.592 : 0.581	0.118	0.731
ACG	0.169	0.187 : 0.162	1.061	0.303
ATC	0.127	0.118 : 0.130	0.312	0.576
ACC	0.120	0.103 : 0.127	1.29	0.256

**Table 6:** Comparison of haplotype frequencies for the analysed *AVPR1b* polymorphisms between patients with suicide attempts and the control group.

Haplotype	Haplotype frequency	Case : control ratio	*χ* ^2^	*P*
Block 1				
AGG	0.823	0.814 : 0.826	0.291	0.589
GGG	0.076	0.068 : 0.079	0.547	0.459
GCA	0.060	0.081 : 0.052	4.087	0.043*
GGA	0.041	0.037 : 0.042	0.162	0.687

*Not significant after correction for multiple test using 10.000 permutations (*P* = 0.130).

**Table 7:** Multilocus interaction model for the risk of suicide attempts with the *NR3C1. AVPR1b* and *CRHR1 *genes by the MDR method.

Model	Loci combination	Testing balanced accuracy (%)	Cross-validation consistency (%)	*P* value
2-locus	Rs258813 of *NR3C1* and rs110402 of *CRHR1 *	47	30	0.989
3-locus	Rs41423247 and rs258813 of *NR3C1* and rs110402 of *CRHR1 *	52	50	0.760
4-locus	Rs41423247 and rs258813 of *NR3C1*. rs28373064 of AVPR1b and rs110402 of *CRHR1 *	50	90	0.589
